# Factors Associated With Post-traumatic Stress Disorder Among Nurses During COVID-19

**DOI:** 10.3389/fpsyg.2022.745158

**Published:** 2022-01-31

**Authors:** Hu Jiang, Nanqu Huang, Weiyan Tian, Shangpeng Shi, Guanghui Yang, Hengping Pu

**Affiliations:** ^1^Nursing Department, The Third Affiliated Hospital of Zunyi Medical University, The First People’s Hospital of Zunyi, Zunyi, China; ^2^School of Nursing, Philippine Women’s University, Manila, Philippines; ^3^Drug Clinical Trial Institution, The Third Affiliated Hospital of Zunyi Medical University, The First People’s Hospital of Zunyi, Zunyi, China; ^4^Quality Control Department, The Third Affiliated Hospital of Zunyi Medical University, The First People’s Hospital of Zunyi, Zunyi, China

**Keywords:** post-traumatic stress disorder (PTSD), nurses, COVID-19, Chinese nurses, post-traumatic growth (PTG)

## Abstract

**Objective:**

To investigate post-traumatic stress disorder (PTSD), perceived professional benefits and post-traumatic growth (PTG) status among Chinese nurses in the context of the COVID-19 pandemic and to compare the differences between nurses working inside and outside Hubei.

**Methods:**

From February 18 to February 25, 2020, the authors constructed the questionnaire using the Questionnaire Star platform, and convenience sampling was used to distribute the questionnaire *via* WeChat. Nurses who worked at the hospital during the COVID-19 pandemic were the research subjects.

**Results:**

A total of 3,419 questionnaires were received, of which 2,860 nurses were working outside Hubei Province and 559 nurses were working inside Hubei Province. Both groups were exposed to COVID-19. The results indicated that gender, job title, department, average monthly income, the number of night shifts per month, hospital classification, specialization, and previous experience with assisting during disasters were statistically significant. The *t*-test results showed that the difference in perceived professional benefits scores between the two groups was not statistically significant, and the differences in PTG scores and PTSD scores between the two groups were statistically significant. The chi-square test indicated that the difference in PTSD prevalence between nurses working outside and inside Hubei Province was statistically significant and that the PTSD prevalence of nurses working outside Hubei Province was higher than that of nurses working inside Hubei Province. One-way ANOVA and independent sample *t*-test results showed that age, job title, job assignment, length of service, average monthly income, number of night shifts per month, number of children, and the Nurses’ Perceived Professional Benefits Scale (NPPBS) and post-traumatic growth inventory (PTGI) scores influenced the prevalence of PTSD. Logistic regression showed that the occurrence of PTSD was associated with average monthly income, length of service, number of children, and the PTGI and NPPBS scores.

**Conclusion:**

During the COVID-19 pandemic, nurses working outside Hubei Province reported greater PTSD than those working inside Hubei Province. The occurrence of PTSD was related to average monthly income, length of service, number of children, and the PTGI and NPPBS scores.

## Introduction

In recent years, the incidence of infectious disease has increased as new pathogens have been discovered, collectively creating enormous challenges for society regarding disease control (Esposito, 2016). Infectious diseases result in severe socioeconomic loss and a high mortality rate, effects especially associated with SARS (Zhong and Zeng, 2006), Ebola (Malvy et al., 2019), and the H7N9 virus, all of which emerged in the 21st century (Tanner et al., 2015). In December 2019, a novel coronavirus disease (coronavirus disease 2019, abbreviated as COVID-19) first emerged in Wuhan, became a large-scale epidemic in China, and subsequently became a global pandemic and the epicenter of public health concerns worldwide.

Public health emergencies require coordination among different personnel at a large scale, and participants, particularly medical personnel, are subjected to high stress levels that could easily lead to psychological disorders. A survey during the SARS pandemic suggested that the high level of stress, anxiety and depression faced by medical personnel may have caused a long-term psychological impact (Preti et al., 2020). The latest studies show that medical personnel exposed to COVID-19 have a higher incidence of depression and anxiety, and the symptoms are severe in particularly nurses (Lai et al., 2020). Mainly medical personnel, among whom nurses comprise the largest proportion, have been managing and containing COVID-19. The intense and highly controlled working environment has created extreme physical and psychological challenges for nurses. Variations in working intensity and the working environment while providing disaster assistance have made work more challenging for nurses. Previous research has focused on mainly the negative impact of the high volume of public health demands on nurses, with less attention given to the growth of nurses while providing disaster assistance (Preti et al., 2020; Feingold et al., 2022).

Post-traumatic growth (PTG) and post-traumatic stress disorder (PTSD) are the most common post-traumatic psychological reactions. PTSD refers to a group of characteristic and persistent symptoms that appear after an individual experiences an unusual threat or traumatic stress event (Iranmanesh et al., 2013). Compared with PTSD, PTG refers to the positive psychological changes that individuals experience after experiencing life crises (Silverstein et al., 2018). Several studies illustrate that medical personnel could acquire PTG from providing disaster assistance, and the extent of this growth seems to be positively correlated with the PTSD score (Nishi et al., 2016). Professional benefit is defined as the satisfaction and the sense of worth that one receives from one’s professional work (Rnn and Dolly Goldenberg, 2008). In China, nurses have a low social status. Chinese culture has improved the social status of nurses during the COVID-19 pandemic. Whether this improvement benefits the entire nurse population remains unclear.

In December 2019, COVID-19 emerged and spread in Hubei Province, China (Wang C. et al., 2020). To cope with the pandemic, thousands of nurses across China traveled to Hubei to provide medical assistance (Chen S. H. et al., 2021). Many studies have examined the mental health and PTG of nurses exposed to COVID-19 (Huang et al., 2020; Sheng et al., 2020; Peng et al., 2021), but few have compared the outcomes with nurses working outside Hubei Province. Therefore, the objectives of this research were to investigate and compare the difference of PTSD, professional benefits and PTG status among Chinese nurses working inside and outside Hubei in the context of the COVID-19 pandemic.

## Materials and Methods

### Sample Size Estimation

The sample size was calculated for a cross-sectional survey. The initial sample size of 2,750 was estimated using the formula *N* = Zα^2^ × p × q/(d^2^), where α (alpha) is the probability of a type I error, p × q is the maximum possible estimate of variance, and d (delta, δ) is the permissible error (Mathew et al., 2021). To account for missing answers in the questionnaire and non-responses, we added 20% to the estimated sample; thus, the estimated sample size needed was 3,300.

### Design and Participants

From February 18 to February 25, 2020, Wenjuanxing^1^, which is a widely accepted online questionnaire survey platform in China for data collection, was used to construct the questionnaire. It was distributed *via* a popular social networking platform called WeChat. The questionnaire was distributed *via* the convenience sampling method by personal contact. Nurses who worked at the hospital during the COVID-19 pandemic were considered the research subjects. The inclusion criteria were nurses who possessed a qualification certificate and were on active duty during the COVID-19 pandemic. The exclusion criteria were nurses who were engaged in further academic study, in the process of standardization training, interning, or had a medical history of PTSD. Informed written consent was obtained from survey participants prior to completing the questionnaires.

### Data Collection and Quality Control

Panel discussion meetings were convened, and the comprehensiveness, accuracy and feasibility of the questionnaires were ascertained and verified *via* multiple stages of amendments. Before the formal survey was conducted, experts provided standardization trainings to the surveyors about the survey procedures, the application of survey tools and the division of work. The Questionnaire Star platform allows limits to be set on answers. All survey items were required to be answered before submission. Therefore, all of the returned questionnaires were valid, and the quality of the questionnaires was controlled. After the survey was completed, the authors reviewed the collected questionnaires.

## Measurement

### The General Information Questionnaire

The research objectives and context were designed to flexibly account for general demographical and sociological data. The data were collected using a self-designed questionnaire. Items included age, sex, highest education level, job title, job assignment, department group, length of service, average monthly income, number of night shifts worked per month, employment status, marital status, number of children, hospital classification, specialization and previous involvement in disaster assistance.

### The Questionnaire for Perceived Professional Benefits

The Nurses’ Perceived Professional Benefits Scale (NPPBS) was derived from the Professional Benefit Scale prepared by Hu and Liu (2013), which includes 33 items and 5 dimensions: positive career perception, nurse–patient relationship, self-development, family acknowledgment, and sense of belonging to the team. Each item is scored *via* a five-point Likert scale that ranges from 1 (very unsatisfied) to 5 (very satisfied). The total score ranges from 33 to 165, and the higher the score is, the higher the perceived professional benefits. The Cronbach’s α for the questionnaire was 0.958, and the Cronbach’s α of each dimension varied between 0.821 and 0.893.

### Post-traumatic Growth Inventory

The post-traumatic growth inventory (PTGI) was derived from that prepared by Tedeschi and Calhoun (1996). The PTGI contains 5 dimensions: interpersonal relationships, personal strength, new possibilities, appreciation of life and spiritual changes, for a total of 21 items. The PTGI is a six-point Likert scale ranging from (not at all) to 5 (very much), with a total possible score ranging from 0 to 105 points. The higher the score is, the better the level and capability of the PTGI. We used the version of the inventory revised by Ji et al. (2011). The *Cronbach’s* α was 0.874.

### Post-traumatic Stress Disorder Scale

The PTSD Scale: Civilian Version (PCL-C) was derived from the fourth edition of the Diagnostic and Statistical Manual of Mental Disorders published by the US PTSD Research Center and is used to assess individuals’ experience of psychological trauma under non-state-of-war conditions. The scale consists of 17 items to evaluate three primary symptoms of PTSD: repeated experience, emotional numbing, and hypervigilance. The PLC-C is frequently used for PTSD diagnosis, intervention, and treatment effect evaluation; it is known for its credibility and efficacy and is one of the most widely applied tools. Each scale item is measured by a score ranging from 1 to 5, with a total possible score ranging from 17 to 85. Several studies use a score of 38 as the threshold value, which effectively and efficiently differentiates PTSD and non-PTSD patients; hence, it can be used to evaluate and identify PTSD (Dobie et al., 2002). A threshold of 38 chosen in this research for the PCL-C, whereby a score greater than 38 indicates that an individual has PTSD, while a score less than 38 indicates that an individual does not have PTSD. The PCL-C scale is widely used in China, and its *Cronbach’s* α is 0.94 (Zhu et al., 2021).

### Ethical Considerations

The study adhered to all ethical principles for the acceptable research conduct with humans outlined by the Declaration of Helsinki. This study was approved by the Ethics Committee of the First People’s Hospital of Zunyi (2020-048), and online informed consent was provided by all survey participants prior to their enrollment. Participants could terminate the survey at any time. The survey was anonymous, and confidentiality of information was assured.

### Statistical Analysis

We analyzed the data using SPSS18.0. We conducted preliminary analyses before formal statistical analyses to avoid common method bias. Categorical data were described by instance, and percentages were tested *via* the chi-square test. Quantitative data were tested using the *t*-test, and the variables that remained statistically significant in univariate tests were subsequently used as inputs in logistic regression analysis. A stepwise method was applied to select the optimal model. Statistical significance was set at *P* < 0.05.

## Results

### Preliminary Analysis

We conducted Harman’s single-factor test with all the measurement items, and the results showed that the primary factor component was approximately 18.90%, which proved that there was no common method bias in our study.

### Demographical Differences Between the Two Groups of Nurses

A total of 3,419 questionnaires were received, for an effective recovery rate of 100%. A total of 3,149 nurses participated in the survey, with 559 (16.35%) nurses who were involved in managing COVID-19 within Hubei Province and 2,860 (83.65%) uninvolved nurses outside Hubei Province. The chi-square test was used to compare the differences in demographics between the two groups of nurses, and the results showed that sex, job title, department, average monthly income, the number of night shifts per month, hospital classification, specialization, and previous experience with disaster assistance were statistically significant (*P* < 0.05) (Table 1).

**TABLE 1:** Basic information analysis.

Variable	Nurses working outside Hubei Province (*n* = 2860)	Nurses working within Hubei Province (*n* = 559)	Δ^2^/*t*	*P*
Age	30.39 ± 5.95	30.32 ± 5.52	0.812	0.065
**Sex**				
Female	2756 (96.4%)	504 (90.2%)	40.571	< 0.001
Male	104 (3.6%)	55 (9.8%)		
**Highest education level**				
Technical secondary school	67 (2.3%)	6 (1.1%)	6.572	0.087
Junior college	992 (34.7%)	184 (32.9%)		
Bachelor’s	1798 (62.9%)	367 (65.7%)		
Master’s and above	3 (0.1%)	2 (0.4%)		
**Job title**				
Entry level	2376 (83.1%)	450 (80.5%)	8.671	0.013
Intermediate level	410 (14.3%)	102 (18.2%)		
Senior level	74 (2.6%)	7 (1.3%)		
**Job assignment**				
Yes	2174 (76.0%)	406 (72.6%)	2.892	0.089
No	686 (24.0%)	153 (27.4%)		
**Department group**				
Department of internal medicine	795 (27.8%)	213 (38.1%)	60.537	< 0.001
Department of surgery	877 (30.7%)	144 (25.8%)		
Department of obstetrics and gynecology	282 (9.9%)	23 (4.1%)		
Department of pediatrics	253 (8.8%)	29 (5.2%)		
Emergency room/ICU	433 (15.1%)	120 (21.5%)		
Other	220 (7.7%)	30 (5.4%)		
**Length of service**				
≤5 years	1216 (42.5%)	233 (41.7%)	0.157	0.925
6–15 years	1361 (47.6%)	271 (48.5%)		
≥16 years	283 (9.9%)	55 (9.8%)		
**Average monthly income**				
≤6000 yuan	2409 (84.2%)	437 (78.2%)	14.093	0.001
6001∼9000 yuan	415 (14.5%)	108 (19.3%)		
>9001 yuan	36 (1.3%)	14 (2.5%)		
**Number of night shifts per month**				
None	902 (31.5%)	121 (21.6%)	25.731	< 0.001
1–3	257 (9.0%)	43 (7.7%)		
≥4	1701 (59.5%)	395 (70.7%)		
**Employment status**				
Affiliate staff	837 (29.3%)	168 (30.1%)	0.279	0.870
Contractor	1819 (63.6%)	354 (63.3%)		
Temporarily employed	204 (7.1%)	37 (6.6%)		
**Marital status**				
Married	2098 (73.4%)	388 (69.4%)	6.638	0.084
Unmarried	690 (24.1%)	154 (27.5%)		
Widowed	2 (0.1%)	2 (0.4%)		
Divorced	70 (2.4%)	15 (2.7%)		
**Number of children**				
None	910 (31.8%)	197 (35.2%)	2.978	0.395
1	1151 (40.2%)	211 (37.7%)		
2	782 (27.3%)	149 (26.7%)		
3 and above	17 (0.6%)	2 (0.4%)		
**Hospital classification**				
Primary	38 (1.3%)	8 (1.4%)	38.744	< 0.001
Secondary	1882 (65.8%)	291 (52.1%)		
Tertiary	940 (32.9%)	260 (46.5%)		
**Specialized nurse**				
Yes	715 (25.0%)	181 (32.4%)	13.167	< 0.001
No	2145 (75.0%)	378 (67.6%)		
**Previous involvement in disaster assistance**				
Yes	267 (9.3%)	193 (34.5%)	254.824	< 0.001
No	2593 (90.7%)	366 (65.5%)		

### Comparison of Scores Between the Two Groups

The results of the independent sample *t*-test analysis indicated that the difference in perceived professional benefits between nurses working outside Hubei Province and nurses working inside Hubei Province was not statistically significant (*t* = −1.562, *P* = 0.118), but the difference in the PTG scores between the two groups was statistically significant (*t* = −2.179, *P* = 0.029). The difference in the PTSD total scores between the two groups was statistically significant (*t* = 3.380, *P* = 0.001) (Table 2).

**TABLE 2:** Comparison of scores between the two groups (*n*,*$\bar{x}$* ± *s*).

Item	Nurses working outside Hubei Province (*n* = 2860)	Nurses working within Hubei Province (*n* = 559)	*t*	*P*-value
NPPBS	138.6 ± 20.7	140.1 ± 21.3	–1.562	0.118
PTGI	95.27 ± 18.07	97.09 ± 18.47	–2.179	0.029
PTSD	42.88 ± 16.69	40.17 ± 17.48	3.380	0.001

### Comparison of Post-traumatic Stress Disorder Prevalence Between the Two Groups

Chi-square tests indicated that the difference in PTSD prevalence between nurses working outside Hubei Province and nurses working inside Hubei Province was statistically significant (χ^2^ = 10.721, *P* < 0.001), with 55.8% of nurses working outside Hubei Province having PTSD and 48.3% of nurses working inside Hubei Province having PTSD. The total PTSD prevalence rate among the survey respondents was 54.6% (Table 3).

**TABLE 3:** Comparison of PTSD prevalence between the two groups.

Variable	Nurses working outside Hubei Province (*n* = 2860)	Nurses working within Hubei Province (*n* = 559)	χ^2^	*P*-value
PTSD-negative	1263 (44.2)	289 (51.7)	10.721	<0.001
PTSD-positive	1597 (55.8)	270 (48.3)		

### Influencing Factors of Post-traumatic Stress Disorder

One-way ANOVA and independent sample *t-*tests were used to analyze which factors influenced whether nurses had PTSD. The results showed that age, job title, job assignment, length of service, average, monthly income, number of night shifts per month, number of children, and the NPPBS and PTGI scores influenced whether nurses had PTSD (Table 4).

**TABLE 4:** Influencing factors of PTSD.

Variable	χ^2^/*t*	*P*
Age	2.809	0.005
Job title	4.845	0.028
Job assignment	0.342	0.004
Length of service	6.892	0.009
Average monthly income	10.195	0.001
Number of night shifts per month	0.531	0.011
Number of children	5.870	0.015
NPPBS score	9.802	< 0.001
PTGI score	4.698	< 0.001

### Multivariate Analysis of Post-traumatic Stress Disorder

Logistic regression was used in multivariate analysis of the PTSD scores. The results showed that the prevalence of PTSD was associated with average monthly income, length of service, number of children, and the PTGI and NPPBS scores (Table 5).

**TABLE 5:** Multivariate analysis of PTSD.

Independent variables	*B*	*SE*	*Wald*	*P*	OR	*95%CI*
Average monthly income	–0.154	0.088	3.112	0.078	0.857	0.722, 1.017
Length of service	–0.147	0.064	5.196	0.023	0.863	0.761, 0.980
Number of children	0.205	0.051	16.323	< 0.001	1.227	1.111, 1.356
NPPBS score	–0.021	0.003	69.012	< 0.001	0.979	0.975, 0.984
PTGI score	0.006	0.003	5.071	0.024	1.006	1.001, 1.011
Constant	2.446	0.264	86.090	< 0.001	11.542	

## Discussion

In this study, we discovered that during the COVID-19 pandemic, the prevalence of PTSD among Chinese nurses was high. The overall prevalence rate of PTSD among nurses was 54.6%. A total of 55.8% of nurses working outside Hubei Province had PTSD, and 48.3% of nurses working inside Hubei Province had PTSD. Our research indicated a high rate of PTSD, which could be attributed to the fact that COVID-19 first emerged in China. Additionally, nurses’ lack of experience with pandemics combined with their direct exposure to COVID-19 patients led to considerable physical and psychological stress. The reported incidence of PTSD in various populations has been high, with an incidence rate between 4 and 41% in the general population (Torales et al., 2020). The survey of Wang Y. X. et al. (2020) suggested that the incidence rate of PTSD among nurses was 16.83%. A Korean study stated that 36.7% of nurses were at risk of developing PTSD (Moon et al., 2021), which was significantly higher than our results. Most importantly, our study compared the prevalence of PTSD among nurses working within and outside Hubei during the early period of the COVID-19 pandemic. The prevalence of PTSD in our survey was higher than in other studies, possibly due to objective factors such as differences in measurement instruments and when the survey was distributed. Furthermore, the prevalence of PTSD among nurses working within Hubei Province was lower than that of nurses working outside Hubei Province. The reason may be that nurses who were involved in COVID-19 were carefully selected and trained rigorously; hence, their level of work engagement may have improved their ability to cope with stress.

Because of individuals’ scopes of knowledge and different work and social experiences, some nurses may tend to focus more on the negative reports of COVID-19. Specifically, married nurses may have been worrying about the safety of their family members while risking exposure to COVID-19; thus, their negative emotions could exacerbate their psychological stresses. As one of the essential personnel in containing pandemics, nurses offer assistance at the first instance of a crisis. As such, intense and repetitive exposure to traumatic events may lead nurses to worry about personal safety, which generates a sense of powerlessness, anxiety, depression or more severe mental disorders such as PTSD (Auxéméry, 2018).

Because of the political context, nurses who embarked to assist Hubei perceived their actions with a sense of obligation and responsibility, and their work caused feelings of honor and pride. In the past, nurses had a low social status (Feng et al., 2017); however, this situation has changed since the outbreak of COVID-19. Not only China but also all of society now perceive nurses more highly, which has affected nurses’ perceived professional benefits to a certain extent. Our findings suggested that the perceived professional benefits of nurses working outside Hubei Province were not significantly different from those of nurses working within Hubei Province (*P* > 0.05). Judging from the values of the results, however, the perceived professional benefits of nurses working inside Hubei Province were higher than those of nurses working outside Hubei Province (140.1 ± 21.3 vs. 138.6 ± 20.7). Few studies have investigated the effect of the COVID-19 pandemic on nurses’ perceived professional benefits, but our study showed that Chinese nurses’ perceived professional benefits improved during the COVID-19 pandemic.

Our results showed that nurses working inside Hubei Province had higher PTG scores than nurses working outside Hubei Province (*P* < 0.05), which implied that nurses inside Hubei Province attained more personal growth throughout their experiences in disease management and containment due to their proactive response. Our results were consistent with several studies that reported that nurses experienced PTG (Chen R. et al., 2021; Cui et al., 2021). However, the PTGI scores in our study were higher than those in other studies. In addition, our results suggested that nurses working inside Hubei Province obtained more growth. Contemporary research results have indicated that PTSD is correlated with PTG (Turner et al., 2018; Magid et al., 2019). However, there was less evidence to support correlation between PTSD and PTG for healthcare professionals (Hong et al., 2021; Feingold et al., 2022). Therefore, more research is needed to further investigate the relationship between PTSD and PTG.

Previous studies have identified multiple sources of PTSD, such as age, sex, job title, social support, exposure intensity and psychological factors (Shrestha, 2015; Šagud et al., 2017; Cirino and Knapp, 2019; Wang Y. X. et al., 2020). Occupation may be a factor in the prevalence of PTSD. For example, several studies report that the nurse population is highly susceptible to PTSD (Salmon and Morehead, 2019). In our research, multivariate analysis indicated that PTSD prevalence was associated with several work-related and sociodemographic variables. Length of service, number of children, and the NPPBS and PTGI scores were independent predictive factors (*P* < 0.05). Length of service and NPPBS score controlled the prevalence of PTSD (*OR* < 1). In contrast to previous studies, our research did not identify heterogeneity between the sexes, which might be due to a lack of male nurses in the sample. The preceding factors directly determined an individual’s work experience and knowledge of disease, which was consistent with international research outcomes (Mealer et al., 2017). In general, it is important to protect nurses’ physical and psychological health. Research has demonstrated that training, experience, perceptiveness, social support and effective coping strategies are key factors in preventing physical and psychological disorders; thus, improving nurses’ ability requires a combination of different approaches (Brooks et al., 2020).

## Limitations

In this study, the data were collected within the Chinese context on a large sample of nurses. The prevalence of PTSD was compared between nurses working inside and outside Hubei Province. Moreover, we analyzed factors associated with PTSD. However, this study had several limitations. First, this was a cross-sectional study. Second, our survey subjects were Chinese nurses, whose cultural context may cause their responses to differ from nurses who work in other countries. The results of this survey represented only the situation in China. In addition, the possibilities that our survey participants were involved in COVID-19 nursing at different time periods, their levels of exposure were different, and the fact that our survey was self-reported could increase the heterogeneity of the research. Lastly, we did not follow-up with participants, and we were unable to verify the relationship between exposure time and the PTSD prevalence rate.

## Practical Implications

The pandemic has caused a global disaster, and mainly nurses are fighting the pandemic. Healthcare managers and nursing supervisors should pay attention to their mental health while avoiding influence by nurses’ mental health disorders. In particular, psychological trauma is caused by long-term and continuous work. Increasing the number of nurses, reducing shifts, improving remuneration, and conducting psychological interventions are necessary and critical methods to prevent the loss of nurses, burnout and psychological trauma.

## Conclusion

Our research found that nurses working outside the Hubei Province reported greater rates of PTSD than those working inside the Hubei Province. The occurrence of PTSD was related to PTG, professional benefits, monthly average income, job title, age, and the number of children. Nevertheless, for future pandemic control and management, efforts should be made to administer psychological intervention to medical personnel to improve their coping abilities.

## Data Availability Statement

The original contributions presented in the study are included in the article/Supplementary Material, further inquiries can be directed to the corresponding author.

## Ethics Statement

The studies involving human participants were reviewed and approved by the Ethics Committee of the First People’s Hospital of Zunyi. The patients/participants provided their written informed consent to participate in this study.

## Author Contributions

All authors listed have made a substantial, direct, and intellectual contribution to the work, and approved it for publication.

## Conflict of Interest

The authors declare that the research was conducted in the absence of any commercial or financial relationships that could be construed as a potential conflict of interest.

## Publisher’s Note

All claims expressed in this article are solely those of the authors and do not necessarily represent those of their affiliated organizations, or those of the publisher, the editors and the reviewers. Any product that may be evaluated in this article, or claim that may be made by its manufacturer, is not guaranteed or endorsed by the publisher.

## Footnotes

1
www.wjx.cn
